# Organometallic Iron(III)-Salophene Exerts Cytotoxic Properties in Neuroblastoma Cells via MAPK Activation and ROS Generation

**DOI:** 10.1371/journal.pone.0019049

**Published:** 2011-04-29

**Authors:** Kyu Kwang Kim, Rakesh K. Singh, Robert M. Strongin, Richard G. Moore, Laurent Brard, Thilo S. Lange

**Affiliations:** 1 Molecular Therapeutics Laboratory, Program in Women's Oncology, Department of Obstetrics and Gynecology, Women and Infants' Hospital, Alpert Medical School, Brown University, Providence, Rhode Island, United States of America; 2 Department of Chemistry, Portland State University, Portland, Oregon, United States of America; 3 Division of Gynecologic Oncology, Department of Obstetrics and Gynecology, Southern Illinois University School of Medicine, Springfield, Illinois, United States of America; 4 Department of Molecular Biology, Cell Biology, and Biochemistry, Brown University, Providence, Rhode Island, United States of America; University of Minnesota, United States of America

## Abstract

The objective of the present study was to investigate the specific effects of Iron(III)-salophene (Fe-SP) on viability, morphology, proliferation, cell cycle progression, ROS generation and pro-apoptotic MAPK activation in neuroblastoma (NB) cells. A NCI-DTP cancer screen revealed that Fe-SP displayed high toxicity against cell lines of different tumor origin but not tumor type-specificity. In a viability screen Fe-SP exhibited high cytotoxicity against all three NB cell lines tested. The compound caused cell cycle arrest in G1 phase, suppression of cells progressing through S phase, morphological changes, disruption of the mitochondrial membrane depolarization potential, induction of apoptotic markers as well as p38 and JNK MAPK activation, DNA degradation, and elevated generation of reactive oxygen species (ROS) in SMS-KCNR NB cells. In contrast to Fe-SP, non-complexed salophene or Cu(II)-SP did not raise ROS levels in NB or SKOV-3 ovarian cancer control cells. Cytotoxicity of Fe-SP and activation of caspase-3, -7, PARP, pro-apoptotic p38 and JNK MAPK could be prevented by co-treatment with antioxidants suggesting ROS generation is the primary mechanism of cytotoxic action. We report here that Fe-SP is a potent growth-suppressing and cytotoxic agent for *in vitro* NB cell lines and, due to its high tolerance in previous animal toxicity studies, a potential therapeutic drug to treat NB tumors *in vivo*.

## Introduction

Neuroblastoma (NB) is the most common extracranial solid tumor and predominantly occurs in children below the age of five. NB accounts for 7–10% of all childhood cancers and in the majority of patients older than 1 year of age the disease is fatal. About 500 new cases of NB are diagnosed in the US each year resulting in 300 deaths annually [1], [2]. Multimodality treatment methods include surgery, radiation therapy, chemotherapy and autologous stem-cell transplantation [3], [4]. These treatment modalities are employed either alone or in combination depending on the location, the biological characteristics of the tumor cells, the stage and the risk group to which the patient belongs. More than 50% of children with high-risk disease will experience a relapse due to drug-resistant residual disease [5], [6]. Eradication of refractory microscopic disease remains one of the most significant challenges in the treatment of the high-risk NB and innovative treatments are needed.

The present report describes the selective cytotoxic effects of organometallic complex Iron(III)-salophene [7] on NB cell lines. In previous reports Fe-SP displayed selective cytotoxicity against ovarian epithelial adenocarcinoma cell lines at concentrations between 100 nM and 1 µM, while the viability of epithelial cervix adenocarcinoma or primary fibroblasts was not affected. Fe-SP treatment of ovarian cancer cells revealed apparent hallmarks of apoptosis, chromatin fragmentation, a loss of mitochondrial transmembrane depolarization potential (ΔΨ_m_), activation of extrinsic and intrinsic apoptosis pathways, exerted effects as an anti-proliferative agent and caused S-phase arrest [8]. Moreover, when injected intraperitoneally in rats, Fe-SP did not show any systemic toxicity at concentrations that revealed chemotherapeutic response in an ovarian cancer cell model *in vivo* [8]. However, the underlying mechanisms by which Fe-SP exerts effects in cancer cells as well as the tumor types that can potentially be targeted remain to be defined. In the present study, we examined activities of Fe-SP against a spectrum of cancer types in a National Cancer Institute-Developmental Therapeutics Program (NCI-DTP) cancer cell growth screen as well as in a viability assay including various NB cell lines. Moreover, we analyzed generation of reactive oxygen species (ROS) by Fe-SP in NB as well as ovarian cancer cells and its impact on activation of apoptotic markers and various mitogen-activated protein kinases (MAPKs).

## Results

### Fe-SP displays differential effects on the viability and growth of various human cancer cell lines

**Figure 1:**
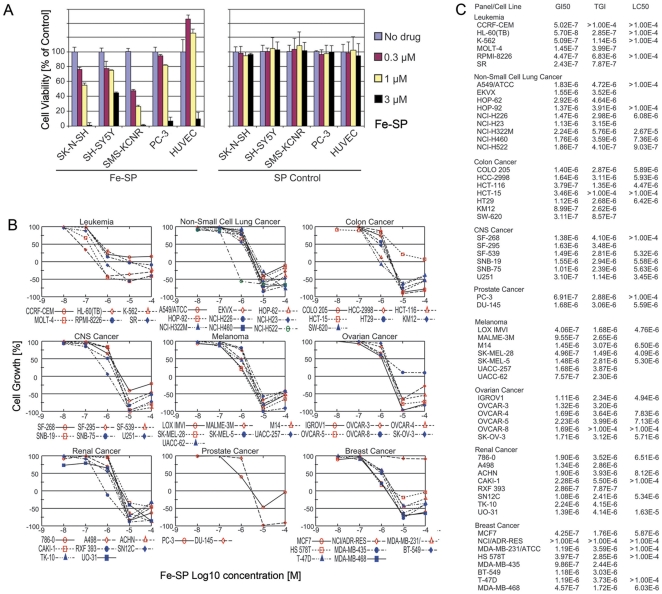
Comparative analysis of the cytotoxic effect of Fe-SP on NB and other cancer cell lines. (**A**) **Viability of NB cell lines upon Fe-SP treatment.** The cytotoxic effect of Fe-SP (0–3 µM) on human NB cell lines (SK-N-SH, SH-SY5Y, SMS-KCNR) was compared to a human cancer cell line of different origin (PC-3) and endothelial cells (HUVEC). Treatment with SP served as a negative control. The MTS viability assay was carried out as described (Materials and Methods). Data are expressed as the mean of the triplicate determinations (X±SD) of a representative experiment in % cell viability of untreated cells [100%]. (**B,C**)** Differential effect of Fe-SP on cell growth in a NCI60 cancer cell line screen.** Fe-SP effects were screened in a NCI60 cell line growth assay (http://dtp.nci.nih.gov/screening.html). Cells were treated in 96 well plates and cell growth of the TCA fixed treated and untreated cells assessed after 48 h.

In an initial approach to analyze the effects of Iron(III)-salophene (Fe-SP) on NB cells we performed a viability assay employing three NB cell lines, SH-SY5Y, parent cell line SK-N-SH and SMS-KCNR. In addition, PC-3 and HUVEC were added to the panel to allow comparison of the effects between NB cells and cells derived from another human tumor or angiogenic cells. The cells were treated for 24 h with various concentrations (0.1–3 µM) of either Fe-SP or non-complexed salophene (SP) as an additional control to untreated controls. SP treatment at ≤3 µM did not affect the viability of any of these cell lines (Fig. 1A). Fe-SP, at 3 µM, exerted high cytotoxic effects on all cells except SH-SY5Y. Remarkably, the response to Fe-SP at concentrations ≤1 µM appeared to be cell type specific with NB cells severely affected, while the effect on prostate cells was less pronounced. Fe-SP at the concentration of 3 µM is similarly cytotoxic to HUVEC cells as compared to tumor cells, but surprisingly at concentrations ≤1 µM Fe-SP stimulated the growth of these endothelial cells. This effect was consistently observed throughout multiple viability assays and should be investigated in future studies.

The NCI-DTP performed a screen on Fe-SP as a growth suppressor against a panel of 60 human cancer cell lines derived from nine tumor types (ovarian, breast, colon, lung, melanoma, leukemia, renal, prostate, central nervous system) (Fig. 1B). The concentration of the drug achieving 50% growth inhibition (GI_50_), total growth inhibition (TGI), and 50% cytotoxicity (LC_50_) was determined by using the dose-response curves with five concentration points of Fe-SP ranging from 10 nM to 100 µM (Fig. 1C). Fe-SP treatment revealed selective growth inhibitory effects against a broad range of cancer cell lines except for NCI/ADR-RES breast cancer cells. Relatively high inhibitory activities by Fe-SP treatment (GI_50_ less than 1.0×10^−6^ M) were achieved against all six leukemia cell lines, 4 of 7 melanoma cancer (LOX IMVI, MALME-3M, SK-MEL-28, UACC-62), 4 of 8 breast cancer (MCF-7, HS 578T, MDA-MB-435, MDA-MB-468), 1 of 2 prostate cancer (PC-3), 3 of 7 colon cancer (HCT-116, KM12, SW-620), 1 of 6 CNS cancer (U251), 1 of 8 renal cancer (RXF 393) and 1 of 9 non-small cell lung cancer (NCI-H522) cell lines. In summary, Fe-SP displayed dose-dependent and selective cytotoxicity depending on the cell line treated.

### Selective morphological changes, disruption of ΔΨ_m_, and induction of apoptosis in NB cells after Fe-SP treatment

**Figure 2:**
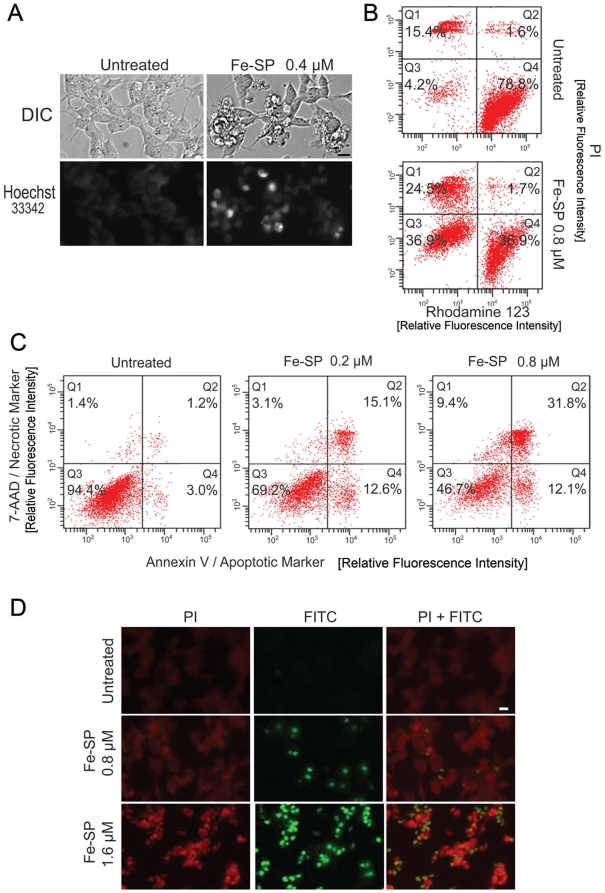
Morphology changes, mitochondrial membrane depolarization potential, apoptotic and necrotic effects and DNA fragmentation in NB cells after Fe-SP treatment. (**A**) **Morphological appearance/DAPI staining.** SMS-KCNR NB cells were treated for 24 h with Fe-SP at a concentration of 0.4 µM before microscopic analysis by DIC or fluorescence analysis after chromatin staining (DAPI) as described (Materials and Methods). Images obtained from a representative experiment are shown. Bar = 10 µm. (**B**) **Mitochondrial membrane depolarization potential (ΔΨ_m_) analysis.** SMS-KCNR NB cells were treated for 24 h with 0.8 µM Fe-SP or SP control, fixed and stained with PI and Rhodamine 123 as described (Materials and Methods). Fluorescence of the single cell population was measured by flow cytometry (right panel) and the transmembrane depolarization potential of the single cell populations plotted. Intact cells = Q4, loss of ΔΨ_m_ = Q3, ruptured cell membrane (and loss of ΔΨ_m_) = Q1 and Q2. (**C**) **Apoptotic and necrotic cell population.** SMS-KCNR NB cells were treated with 0.2 or 0.8 µM Fe-SP or for 24 h and floating and attached cells collected and combined. The quantification of apoptotic cells (Annexin V plasma membrane staining) and necrotic cells (7-AAD DNA staining) of SKOV-3 cells was carried out by flow cytometry as described (Materials and Methods). Viable cells = Q3, necrosis = Q1, early apoptosis = Q4, late apoptosis/necrosis = Q2. (**D**) **Analysis of DNA fragmentation in a TUNEL Assay.** SMS-KCNR NB cells were treated with Fe-SP (0.8, 1.6 µM) for 24 h. A TUNEL assay was carried out by co-staining with fluorescein-12-dUTP (labeling of DNA nicks in apoptotic cells) and of chromatin with propidium iodide (Materials and Methods). During fluorescent microscopy, representative images were taken, apoptotic stain (green) and nuclear stain (red) overlaid. TUNEL positive nuclei due to DNA fragmentation appear as yellow areas. Bar = 10 µM.

To analyze morphological changes of SMS-KCNR NB cells we carried out light (DIC) and fluorescence microscopy of nuclear chromatin staining. Membrane permeable Hoechst 33342 nuclear stain was directly added to the non-permeabilized cells without any fixative. Untreated SMS-KCNR cells displayed a homogenous morphology with nuclei lightly and evenly stained by Hoechst 33342 (Fig. 2A). In contrast, after treatment with 0.4 µM of Fe-SP, SMS-KCNR cells displayed changes in morphology with hallmark features of apoptosis including cell shrinkage, highly condensed and densely stained nuclei in half of the population.

To understand the mechanism(s) involved in the response of NB cells to Fe-SP treatment we examined the mitochondrial transmembrane depolarization potential (ΔΨ_m_) of SMS-KCNR by flow-cytometry. The NB cells were double-stained with PI (chromatin stain in cells with ruptured cell membrane) and Rhodamine 123 which accumulates in mitochondria and directly correlates to the integrity of ΔΨ_m_. The majority of untreated SMS-KCNR cells were viable (Figure 2B, upper panel, Q4) as depicted by uptake of Rhodamine 123 without nuclear PI staining. In contrast, Fe-SP treatment (0.8 µM. 24 h) revealed loss of the ΔΨ_m_ in the majority of cells (Q1 + Q3) with 36.9% of the cells still possessing intact cell membranes (Q3) and 24.5% exhibiting ruptured cell membranes and according loss of ΔΨ_m_ (Q1). The controls with background staining of intact cells by Rhodamine 123 (Q1) indicating a loss of ΔΨ_m_ were comparable for untreated SMS-KCNR (1.6%) and Fe-SP treated cells (1.7%). Loss of ΔΨ_m_ due to chemical agents has been reported to be an indicator of onset of early apoptotic events [9].

To determine the percentage of apoptotic versus necrotic cells after Fe-SP treatment SMS-KCNR cells were treated with 0.2 or 0.8 µM Fe-SP or non-complexed SP for 24 h. Floating and attached cells were collected and combined and flow cytometry performed. The quantification of apoptotic cells was determined by staining with Annexin V, and of necrotic cells by 7-AAD staining. Combination staining with both markers allowed discrimination between early apoptotic cells (Annexin V positive), late apoptotic cells (Annexin V and 7-AAD positive), and necrotic cell death (7-AAD positive). For Fe-SP treated cells, 27.7% and 43.9% were apoptotic with 0.2 and 0.8 µM treatments, respectively, as determined by the combination of cells both in early (Q4) and late apoptosis (Q2) (Figure 2C). In contrast only 4.2% of non-treated cells underwent apoptosis. Necrosis (Q1 + Q2; Figure 2C) was observed for 18.2% (0.2 µM) and 41.2% (0.8 µM) of Fe-SP treated cells (the majority of which were in late apoptotic state; Q2), while necrosis remained at background levels of 2.6% in non-treated cells.

**Figure 3:**
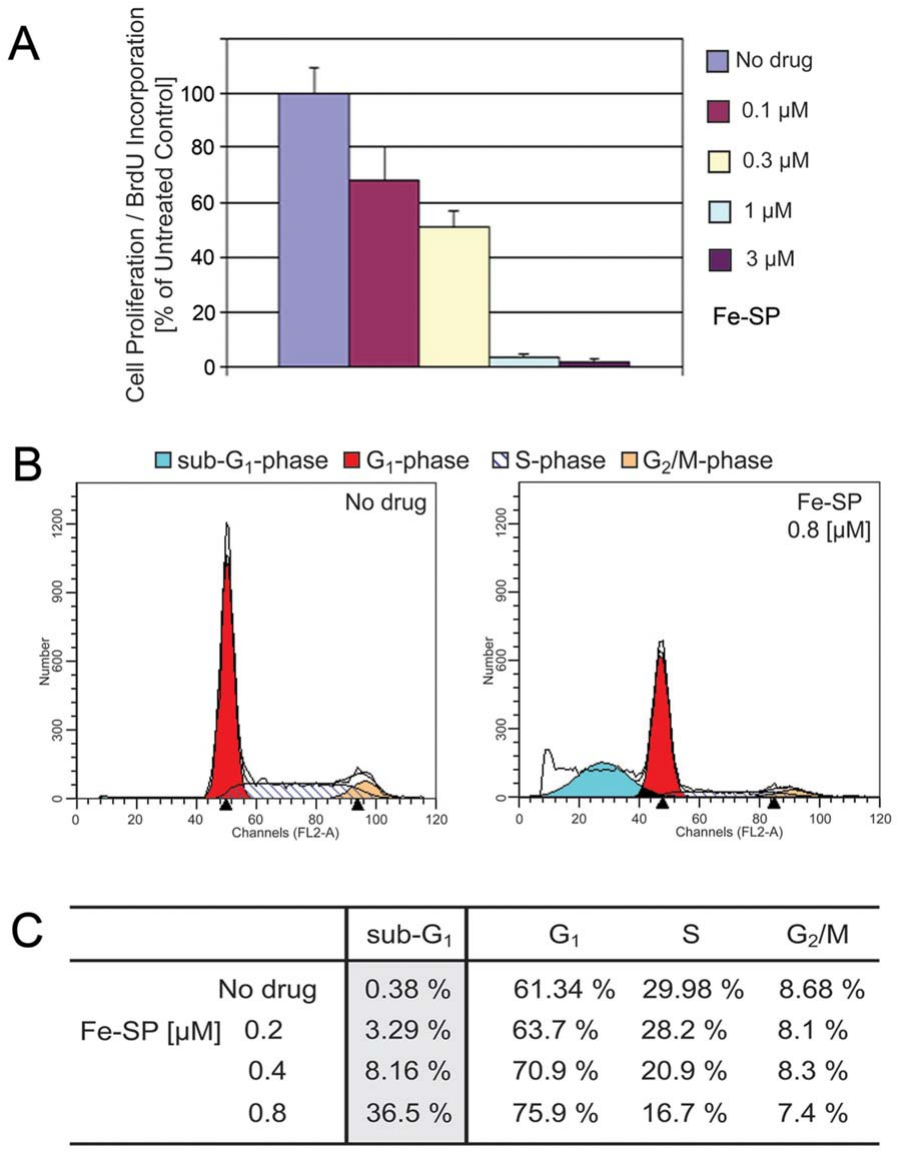
Fe-SP inhibits proliferation of NB cells. (**A**) **BrdU incorporation assay.** SMS-KCNR NB cells were treated with various concentrations (0.1–3 µM) of Fe-SP for 24 h. A colorimetric assay (based on BrdU incorporation) was carried out as described (Materials and Methods). Data are expressed as the mean of the triplicate determinations (X±SD) in % of absorbance by triplicate samples of untreated cells [ = 100%]. (**B,C**) **Fe-SP blocks cell cycle progression in G1 phase.** SMS-KCNR NB cells were treated with 0.2, 0.4 and 0.8 µM Fe-SP for 24. Cell cycle analysis by FACS based on propidium-iodide intercalation into the cellular chromatin was carried out as described (Materials and Methods). Data are presented as (**A**) relative fluorescence intensity in a 2-dimensional FACS profile (ModFit LT software; black lines = data line and model fit line of entire population; shaded areas = model components/subpopulations of G0/G1, S, G2/M, apoptotic cells or in (**B**) a table. Standardized gating was used for all samples. Ten thousand events were analyzed for each sample.

A common method to detect cellular apoptotic events is a TUNEL assay. The assay relies on the presence of nicks in the DNA of apoptotic and some necrotic cells, which can be identified by terminal transferase that will catalyze the addition of labeled dUTP (here: FITC). SMS-KCNR cells were treated with either 0.8 or 1.6 µM Fe-SP for 24 h. To identify cell nuclei, counterstaining with propidium iodide (Pi), which intercalates in DNA, was carried out. TUNEL-positive nuclei were identified by yellow spots resulting from an overlay of the image with apoptotic stain (FITC) and nuclear stain (Pi). As shown (Fig. 3D) no cells before treatment (top panel), a significant portion of the population treated with 0.8 µM (middle panel) and all cells at 1.6 µM Fe-SP (bottom panel) were TUNEL-positive cells indicating fragmented DNA.

### Anti-proliferative effect and cell cycle arrest after treatment of NB cells with Fe-SP

As described in the previous sections Fe-SP is a selective cytotoxic drug in NB cells. To investigate if Fe-SP exerts anti-proliferative effects we performed a BrdU incorporation assay. Fe-SP treatment for 24 h dose-dependently reduced cell proliferation (Fig. 3A) with an IC_50_ value of 300 nM. At the sub-cytotoxic drug concentration of 100 nM Fe-SP BrdU incorporation into DNA was reduced by 48% (Fig. 3A).

In addition to the cell proliferation assay, cell cycle analysis of propidium iodide stained SMS-KCNR cells by flow cytometry was carried out. Fe-SP treatment for 24 h led to an increase in the count of apoptotic sub-diploidal/2n cells (sub-G1, Fig. 3B) in a dose-dependent manner (Fig. 3C). With respect to cycling cells, Fe-SP caused a dose-dependent decrease of cells in S-phase and an increase in G0/G1 phase. 61% of untreated cells are in G0/G1 and 30% are in S phase while treatment with 0.8 µM Fe-SP increased G1 cells to 76% and reduced to 17% in S phase. In summary, upon NB treatment with Fe-SP a developing arrest of cells in G1 and suppression of cells progressing through S phase of the cell cycle was observed.

### Cytotoxicity of Fe-SP or Cu-SP in NB cells, generation of intracellular Reactive Oxygen Species (ROS) and inhibition of cytotoxicity by antioxidant ascorbic acid

Cytotoxic action by organometallic compounds can often be linked to the generation of ROS [10], as shown previously in our lab for HNTMB (not related to salophene) treatment of ovarian cancer cells [11]. Like SP, HNTMB can chelate iron and copper of different oxidation states and as a copper-complex displays properties as an anti-cancer drug and alternative to platinum derivatives in the treatment of various solid tumors. In contrast to HNTMB, SP displays high cytotoxicity to ovarian cancer cells only when bound to iron but not copper [8]. We tested the effect of SP when either complexed as Fe(III)-SP or Cu(II)-SP in NB cell lines and determined the associated generation of ROS.

**Figure 4:**
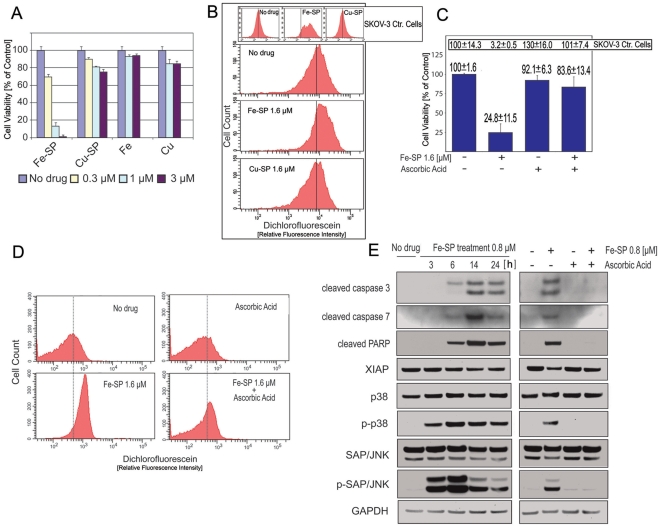
Effect of Fe-SP or Cu-SP on viability, ROS generation, and induction of apoptic markers and MAPK expression. (**A**) **Cytotoxicity of Fe-SP or Cu-SP in NB cells.** The viability assay was carried out after 24 h treatment of SMS-KCNR NB cells with 0–3 µM of Fe-SP, Cu-SP or respective metal salts. Experiments were performed in triplicates; data are expressed as the mean of the triplicate determinations (X±SD) of a representative experiment in % cell viability of untreated cells [ = 100%]. (**B**) **Generation of intracellular Reactive Oxygen Species (ROS) after Fe-SP treatment.** Generation of intracellular ROS following SMS-KCNR NB cells (large panels) or SKOV-3 OC control cells (small panels) after treatment for 4 h with 1.6 µM of Fe-SP or Cu-SP was measured by flow cytometry (see Materials and Methods). Data are presented as relative fluorescence intensity in a 2-dimensional FACS profile. Standardized gating was used for all samples. (**C**) **Fe-SP cytotoxicity is blocked by antioxidant ascorbic acid.** Cells were treated with with1.6 µM Fe-SP alone or in combination with antioxidant ascorbic acid. Viability of SMS-KCNR NB cells is presented as bar diagram and in percentages and of SKOV-3 OC control cells in percentages (insert). (**D**) **Inhibiton of ROS generation in NB cells after Fe-SP treatment.** Generation of intracellular ROS in SMS-KCNR NB was measured after treatment with 1.6 µM Fe-SP alone or in combination with antioxidant ascorbic acid (200 µM). (**E**) **Expression of apoptotic markers and MAPK in NB cells after Fe-SP treatment with and without inhibiton of ROS generation.** SMS-KCNR NB cells were treated with 0.8 µM Fe-SP in the absence (3, 6, 14, 24 h treatment, left panel) or presence of ascorbic acid (200 µM, 24 h treatment, right panel). Immunoblotting was carried out with primary antibodies against PARP-1, caspase-3, -7, pro-survival marker XIAP (inhibitor of effector caspases), and pro-apoptotic MAPK SAP/JNK or p38 in the active (phosphorylated) or inactive form. As an internal standard for equal loading blots were probed with an anti-GAPDH.

SMS-KCNR cells were treated for 24 h with various concentrations (0.1–3 µM) of either Fe-SP or Cu-SP or the respective metal chloride salts alone (FeCl_3_, CuCl_2_) as negative controls. In ovarian cancer cell lines neither Fe^3+^ (≤60 µM) nor Cu^2+^ (≤30 µM) displays significant cytotoxic effects [8]. Accordingly, we did not observe a significant decline of viability in NB cells when treated with the metal chlorine salts (Fig. 4A). In contrast to Fe-SP, which displayed high cytotoxicity even at 300 nM, the cytotoxicity of Cu-SP at 3 µM did not exceed 23% and was only marginally higher than that of the copper-salt alone (Fig. 4A). Next, we determined if SMS-KCNR NB cells and as additional comparison, SKOV-3 ovarian cancer cells, following treatment with Fe-SP or Cu-SP, displayed an increased generation of ROS. Hydrogen peroxide (H_2_O_2_), hydroxyl radicals (HO^•^), and peroxyl radicals (ROO^•^) were detected via Carboxy-H2DCFDA, which is a fluorescein derivative that is cell-permeable and non-fluorescent. In the presence of a cellular oxidant, the molecule is oxidized and produces green-fluorescence that is detected by flow cytometry. As shown in Fig. 4B, ROS generation in NB cells or SKOV-3 cells (insert) remained unchanged as compared to untreated controls when Cu-SP was used. However, ROS generation increased (shift in relative fluorescence intensity, Fig. 4B) following treatment of cells with 1.6 µM Fe-SP (for 4 h) for NB cells as well as for SKOV-3 cells (insert) which correlated with a reduction in cell viability by these compounds at the same concentration (Fig. 4A for NB cells; see reference 8 for SKOV-3 cells).

To determine if generation of ROS by Fe-SP is a major mechanism of cytotoxic action we next performed the viability assay with SMS-KCNR cells or as comparison SKOV-3 cells, treated (for 24 h) with antioxidant ascorbic acid alone or in combination with 1.6 µM Fe-SP. Ascorbic acid is known to inhibit the intracellular accumulation of ROS upon drug treatment in various cell types including neuroblastoma (e.g. treated with fenretinide) [12]. As shown in Fig. 4C (bar diagram), co-treatment with Fe-SP and ascorbic acid almost completely inhibited the cytotoxic effect of the drug in NB cells. While ascorbic acid alone led to a slight reduction of viability (92%) and Fe-SP alone was cytotoxic (25% viability) the combination of both drug and antioxidant restored the viability of NB cells (to 84%). The same observation applies to SKOV-3 cells (Fig. 4C; insert) where ascorbic acid lead to a complete inhibition (viability restored to 101%) of the cytotoxic effect of Fe-SP (viability of 3.6%).

The proof that antioxidants not only inhibit the cytotoxicity of Fe-SP but also block its underlying mechanism of action, i.e., increased generation of ROS, is shown in Fig. 4D. The generation of ROS in SMS-KCNR NB was measured after treatment with 1.6 µM Fe-SP alone or in combination with antioxidant ascorbic acid (200 µM). The ROS generation upon treatment with ascorbic acid alone (negative control) remained unchanged as compared to untreated cells. The ROS generation by NB cells increased (shift in relative fluorescence intensity) following treatment of cells with 1.6 µM Fe-SP for 24 h (positive control), and upon co-treatment ROS generation decreased to nearly the level of the controls (Fig. 4D). In summary, ROS generation induced by Fe-SP in NB cells is the primary mechanism of cytotoxic action.

### Expression of apoptotic markers and MAPK after Fe-SP treatment with and with out inhibition of ROS generation

To define key signaling responses of SMS-KCNR NB cells to treatment with Fe-SP we analyzed by western blot the activation/inactivation of various apoptotic markers such as caspases as well as the expression and activation/phosphorylation of cellular MAPKs involved in pro-apoptotic signaling. Moreover, we analyzed the role of ROS in regulation of these factors and the subsequent onset of cell death by treating cells with antioxidant ascorbic acid alone or in combination with Fe-SP.

Drug treatment leading to programmed cell death (apoptosis) results in the activation of initiator caspases which subsequently activate downstream effector caspases that are responsible for the cleavage of many intracellular proteins, leading to the morphological and biochemical changes associated with apoptosis. Immunoblotting of PAGE-separated cellular lysates revealed that Fe-SP (at 0.8 µM) caused a rapid (within 6 h) and sustained activation/cleavage of effector caspases such as caspase-3 and -7 (Fig. 4E, left panels). These observations were accompanied with the inactivation/cleavage of downstream target PARP which participates in and is a hallmark event of cells undergoing apoptosis. Signals for these three apoptotic markers peaked within 14 h of treatment. The level of pro-survival marker XIAP, a direct inhibitor of effector caspases such as caspase-3, was gradually down-regulated by the treatment of Fe-SP (0.8 µM) in a time-dependant manner. Since the treatment of SMS-KCNR by Fe-SP led to ROS induced cytotoxicity (Fig. 4A–C) we investigated the effect of antioxidant ascorbic acid (at 200 µM) on caspase-3, -7, PARP and XIAP. Inhibition of ROS production completely abolished Fe-SP-induced activation of caspases, inhibited PARP and restored expression of pro-survival marker XIAP to its basal level within the experimental period (as show for 24 h treatment, Fig. 4E right panels). We also investigated the role of ROS induction and the effect of ROS inhibition on the expression and activation of p38 and SAP/JNK in SMS-KCNR cells. Both these MAPKs are crucial factors in signaling cascades responding to inflammatory cytokines, stress, UV light, osmotic shock, cytotoxic drugs and diverse pro-apoptotic stimuli [13]. Fe-SP treatment at 0.8 µM did not significantly affect the level of total p38 or JNK but induced rapid (within 3 h) and sustained phosphorylation of both p38 and SAP/JNK (Fig. 4E, left panels). Activation of these pro-apoptotic markers by Fe-SP was completely blocked by co-treatment of the cells with antioxidant ascorbic acid (Fig. 4E, right panels). In summary, ROS generation induced by Fe-SP in NB cells is the primary mechanism of cytotoxic action and causes induction of apoptosis, reduction of DNA repair mechanisms and activation of pro-apoptotic MAPK.

## Discussion

The structure and synthesis of the novel compound iron-salophene (Fe-SP) and its potency as a growth-suppressing agent on ovarian cancer (OC) cells *in vitro* and in animal models *in vivo* have been previously described [7], [8]. Salophenes represent compounds defined by two Schiff's bases connecting three aromatic moieties that potently bind to transition metals and are closely related to salens that are constituted of aliphatic diamines ([7] and references therein). The present study investigates the cytotoxic, anti-proliferative and apoptotic effect of Fe-SP on various human neuroblastoma (NB) cell lines and defines the excessive generation of ROS as the underlying mechanistic cause of the drugs anti-cancer activity in NB as well as in OC cells.

In an initial approach to analyze the effects of Fe-SP on NB cells we performed a viability assay employing three cell lines: SH-SY5Y neuronal (N)-type cells, their parent cell line SK-N-SH which is MYCN deficient and displays both (N)- and stromal (S)-type NB cells [14], and SMS-KCNR cells which feature MYCN amplification and generally exhibit a uniform phenotype with small, round (N)-type cells that have short neuritic processes [15]. HUVEC (endothelial cells) were included to allow comparison of the effects between NB cells and angiogenic cells. PC-3 cells (prostate adenocarcinoma) were added to the panel as a control since the effect of Fe-SP on these cells has been described previously [8]. More importantly, we desired to define the effect of Fe-SP with respect to tumor origin in a NCI-DTP study against a panel of cell lines which does not include NB cell lines but PC-3 cells, which served as internal standard for the present study. Remarkably, Fe-SP showed high cytotoxicity against all three NB cell lines tested by us while the NCI-DTP screen suggested that Fe-SP in general does display cell line-specificity but not tumor type-specificity (with GI_50_ values at 57 nM Fe-SP for HL-60 leukemia cells but at 0.5 µM for K-562 leukemia cells or >100 µM Fe-SP for ADR-RES breast cancer cells but 0.4 µM for HS 578T breast cancer cells; also see result section). Thus, Fe-SP may be a potential drug to treat a broad range of NB tumor types but not generally be considered as a standard drug against tumors of different origin.

The present state of research in the field of organometallic compounds such as salophenes or salens allows us to only speculate on the possible mechanism(s) of cytotoxic action of Fe-SP in cancer cells. Few data on the effect of transition metal complexes in general on the cell cycle exist, such as the arrest of a neuroblastoma cell line in G1-phase when treated with an isatin-schiff base copper(II) complex [16]. Similarly, few publications examining the change in cell cycle progression following treatment with either a salen (Cr(III)-salen treatment of fibroblasts) [17] or salophene metallocomplex (Fe-SP treatment of OC cells) exist [7]. We show here that Fe-SP dose-dependently reduced the proliferation of NB cells (IC_50_ at 300 nM) even at the minimally cytotoxic concentration of 100 nM. Additional cell cycle analysis of NB cells after Fe-SP treatment revealed a dose-dependent increase in the sub-diploidal population which represents cells with significant DNA damage, indicating a late apoptotic stage which substantiates the finding by the TUNEL assay. Fe-SP at in the range of 200–800 nM also caused dose-dependent arrest of cell cycle progression. The percentage of cells in the G1 subpopulation increased, causing reduction of progression of cells into S-phase while the G2/M population remained similar to untreated controls. Blocking the progression of dividing cells through S-phase reduces the opportunity of DNA repair to counteract drug effect. Future studies could focus on cell cycle checkpoints affected by Fe-SP in synchronized NB cancer cells. Generally, targeting cell cycle regulators has been suggested as a supplemental approach to anti-cancer therapies [18], [19].

A remarkable feature of salens, not yet analyzed for salophenes, is their affinity towards a variety of aromatic neutral molecules [20], [21]. Salens when complexed with transition metals induce DNA scission controlled by the type and charge of the central metal ion core [22]–[24]. Accordingly, for OC cells [7], [8] and in the present report for NB cells we observed high cytotoxicity, DNA fragmentation and induction of apoptosis by the Fe(III)-SP complex. It has been postulated that Fe-salen in cooperation with the quinine system facilitates the formation of O_2_^−^ species to produce free hydroxy radicals responsible for DNA cleavage [25]. The present study shows that the cytotoxic effect and induction of apoptosis by Fe-SP is predominantly mediated by the excessive generation of ROS in cell lines derived from NB or OC. ROS have been implicated in cancer initiation and progression [26], [27]. Cancer cells, presumably through mitochondria dysfunction and increased metabolism, generate a relatively high level of ROS. However, their tolerance to ROS compares to non-transformed cells. Accordingly, further upregulation of cellular ROS, such as shown here after treatment with Fe-SP, has been suggested as a strategy to selectively target cancer cells over normal cells [28]–[30]. Generally, ROS are tightly regulated in balance with cellular defensive antioxidants, such as catalase and SOD, and can participate in a multitude of cellular functions including signal transduction [31].

In the present study, we report that Fe-SP in NB cells induced sustained activation of p38 and JNK, two MAPK mediating cellular signaling pathways in response to inflammatory cytokines, UV light, pro-apoptotic stimuli or cytotoxic drugs. Recent studies proved that the activation of both these pro-apoptotic MAPKs in a ROS-dependent manner mediated the cytotoxic action of chemotherapeutic drugs in cancer cell lines including NB [12], [32]. The present study not only determined that Fe-SP induced ROS generation is the primary mechanism of cytotoxic action but is also responsible for strong activation of p38 and JNK, which can completely be abolished by cellular co-treatment with exogenous antioxidants. Interestingly, several studies have shown that ROS generation is the key mechanism of cytotoxicity for several common chemotherapeutic drugs that are in clinical use or in trials to treat NB which include daunorubicin, cyclophosphamide, cisplatin, fenretinide [12], [33]–[35]. In NB cells fenretinide-mediated ROS induced sustained activation of JNK/p38 MAPK and apoptosis [12] in a similar manner as shown here for relatively low concentrations of Fe-SP. Excessive ROS generation also appears to be linked to cytotoxicity of low Fe-SP doses in OC cells *in vitro* as shown in the present study but Fe-SP *in vivo* does not cause any symptoms of toxicity [8]. In two independent experiments, when rats received Fe-SP intraperitoneally, systemic toxicity at high concentrations (4.0 mg/Kg body weight) was not observed while treatment in an OC rat model revealed chemotherapeutic response even at low concentrations (of 0.5–1 mg/Kg body weight) including complete responses within 12 days of treatment [8].

In summary, the present work, along with our previously published studies [7], [8], suggest that Fe-SP can be developed for the treatment of NB. Potentially, Fe-SP-mediated ROS generation may exert synergistic effects when combined with other agents, thought to modulate the antioxidant functions of cancer cells, for example 2-methoxyestradiol (SOD inhibitor), tetrathiomolybdate or ATN-224 (copper-depletion agents to target Cu/Zn SOD) and buthionine-sulfoximine (inhibitor of glutathione/GSH synthesis). Buthionine-sulfoximine has been intensively investigated and in NB cells potentiated the chemotherapeutic effect of melphalan [36]. In line with these findings, it can also be postulated that the use of different agents leading to GSH depletion (such as isothiocyanate and aziridine analogues) may express therapeutic potential to sensitize cells when combined with ROS-generating chemotherapy through drugs such as Fe-SP.

We suggest future studies to determine the chemotherapeutic effect of Fe-SP in NB animal models as well as the investigation of synergistic effects of Fe-SP with redox-modulating agents. In addition, the role of Fe-SP-induced and ROS-mediated signaling by p38 and JNK MAPK in apoptotic and/or tumorigenic events can be investigated by co-treatment with MAPK inhibitors or growth factors (e.g. EGF) modulating the pro-apoptotic response of NB or OC cells [8], [37].

The present report suggests that Fe-SP is a potent growth-suppressing and cytotoxic agent *in vitro* for NB derived cell lines and a potential therapeutic drug to treat such tumors *in vivo* either alone or in combination with standard therapeutics, cell cycle- or redox-modulating agents.

## Materials and Methods

### Cell Culture

The SMS-KCNR human NB cell line was a gift from Dr. Giselle Saulnier-Sholler (University of Vermont, Burlington, VT). SK-N-SH and SH-SY5Y (human NB), SKOV-3 (human ovarian adenocarcinoma) and PC-3 (prostate adenocarcinoma) were obtained from American Type Culture Collection (Manassas, VA). HUVEC (human umbilical vein endothelial cells) were obtained from Lonza Inc. (Allendale, NJ). SH-SY5Y cells were grown in complete DMEM media containing 10% FBS, 100 units/ml of penicillin, 100 µg/mL of streptomycin, supplemented with 1% non-essential amino acids (Invitrogen catalog#11140). SMS-KCNR and SK-N-SH, cells were maintained in complete RPMI media (10% fetal bovine serum, FBS, 100 units/mL of penicillin, 100 µg/mL of streptomycin). The other cell lines used were cultured according to the providers' recommendations. Cells were maintained at 37°C with 5% CO_2_ in a humidified incubator. For all cell assays, after seeding, cells were allowed to attach overnight in complete medium before treatment. Antioxidant ascorbic acid (Sigma-Aldrich, Saint Lois, MO) was added to some assays at the concentration of 200 µM (SMS-KCNR) or 700 µM (SKOV-3).

### Cell Viability Assay

Viability of Fe-SP or vehicle treated cells was determined by the CellTiter 96 AQueous-One-Solution Assay (Promega, Madison, WI, USA). The assay was carried out as described previously [8] with incubation periods as indicated.

### NCI 60 cell line assay

Fe-SP was screened through the NCI-DTP 60 human cancer cell line panel under the *in vitro* cell line screening project (IVCLSP) as described previously [11] and www.dtp.nci.nih.gov in a colorimetric assay. Percentage growth inhibition was calculated (time zero = Tz; control growth = C, and test growth in the presence of drug at the drug concentration = Ti) as: [(Ti-Tz)/(C-Tz)]×100 for concentrations for which Ti>/ = Tz, [(Ti-Tz)/Tz)]×100 for concentrations for which Ti<Tz the percentage growth was calculated.

### Morphological Studies

SMS-KCNR cells were seeded (1×10^4^/chamber) into a Lab-Tek Chamber-Slide System (Nalge Nunc., Naperville, IL) and treated for 24 h with 0.4 µM Fe-SP alongside with non-treated cells. The cells were fixed in PBS, 2% PFA, 0.2% Triton X for 20 min at RT and stained for 10 min with 200 ng/mL 4′-6-Diamidino-2-Phenylindole (DAPI) in PBS before mounting. Representative images were taken with an inverted microscope (Nikon Eclipse TE2000-E, CCD camera) and 20× objective.

### Analysis of Mitochondrial Transmembrane Depolarization Potential (ΔΨ_m_)

SMS-KCNR cells (1×10^6^) were seeded into 6-well plates and treated with 0.8 µM Fe-SP for 24 h. The cells were incubated with Rhodamine 123 (13 µM) for 30 min at 37°C prior to completion of the drug treatment. Rhodamine 123 is a cationic dye which localizes in the mitochondria of viable cells. The cells were washed, harvested, resuspended in medium containing Propidium Iodide (7.5 µM) and analyzed by flow cytometry. Data was acquired on a BD FACSort flow cytometer using CellQuest software (BD Immunocytometry-Systems, San Jose, CA) and analyzed (ModFit LT software, Verity Software House, Inc., Topsham, ME). Ten thousand cells were analyzed for each sample.

### Determination of Apoptotic and Necrotic Cells (by FACS)

The quantification of apoptotic cells was determined by combination staining with Annexin V and 7-Amino-actinomycin (Apoptosis detection kit; BD Biosciences, San Jose, CA) allowing discrimination between early apoptotic cells (Annexin V positive), late apoptotic cells (Annexin V and 7-AAD positive), and necrotic cell death (7-AAD positive). SMS-KCNR cells were seeded into 100 mm^2^ tissue culture dishes (1×10^6^ cells/dish), treated (24 h, 0.8 µM), floating and attached cells combined, washed once with PBS, pH 7.4, (250×g, 5 min) and stained. Analysis followed immediately on a Becton Dickinson (San Jose, CA) FACSCalibur. Ten thousand events were analyzed for each sample.

### TUNEL Assay

DNA fragmentation was detected using the DeadEndTM Fluorometric TUNEL System assay (Promega, Madison, WI) according to the manufacturer's recommendations. SMS-KCNR cells (15×10^3^/well) were plated into 96 well flat bottom plates (Corning, Inc., Corning, NY), treated with 0.8 or 1.6 µM Fe-SP for 24 h and the assay carried out as described previously [7]. Fluorescence of apoptotic cells (green; labeling of DNA nicks by fluorescein-12-dUTP) and of chromatin (red; staining of chromatin with propidium iodide) was detected by fluorescence microscopy with an inverted microscope (Nikon Eclipse TE2000-E) and a 10× objective. Four randomly chosen microscopic fields were captured.

### Cell Proliferation Assay

Cell proliferation was determined by a BrdU assay (Roche Applied Science, Indianapolis, IN, USA) measuring the incorporation of the pyrimidine analogue, 5-bromo-20-deoxyuridine (BrdU) during DNA synthesis. SMS-KCNR (15×10^3^) were seeded into 96-well flat bottom plates (Corning Incorporated, Corning, NY, USA) before treatment with Fe-SP (0–3 µM) for 24 h. The assay was carried out as described previously [37].

### Cell Cycle Analysis

Cell cycle analysis and quantification of apoptosis was carried out by flow cytometry. SMS-KCNR (5×10^5^) cells were seeded into 6-well culture plates (Corning Inc., Corning, NY) and treated under the condition as indicated. After 24 h cells were collected, fixed and stained with propidium iodide (100 µg/mL) in PBS containing sodium citrate (1 mg/mL), Triton-X-100, and RNAse (20 µg/mL) for 30 min. Data was acquired on a BD FACSort flow cytometer using CellQuest software (BD Immunocytometry Systems, San Jose, CA) and analyzed by using ModFit LT software (Verity Software House, Inc., Topsham, ME). Ten thousand events were analyzed for each sample. Appropriate gating was used to select the single cell population and used on all samples, ensuring that the measurements were made on a standardized cell population.

### Western Blot Analysis

SMS-KCNR cells (1×10^6^) were seeded into 100 mm^2^ tissue culture dishes and treated with Fe-SP as indicated. The cells were scraped off and lysed on ice with Cell Extraction Buffer (BioSource International, Inc., CA.) supplemented with a protease inhibitor cocktail and phenylmethylsulfonyl fluoride (Sigma-Aldrich, MO) according to the manufacturers' recommendations. After centrifugation, the supernatant was kept and Bio-Rad DC Protein Assay kit (Hercules, CA.) was used to quantify protein concentrations. Protein electrophoresis was performed by using the Xcell SureLockTM mini-cell electrophoresis system in a NuPAGE 4–12% Tris-Bis Gel in NuPAGE MES SDS running buffer, transferred onto a PVDF membrane, blocked with 5% nonfat dry milk in PBS-Tween and probed against primary antibodies (cleaved Caspase 3 #9661, cleaved Caspase 7 #9491, cleaved PARP #9541, XIAP #2045, P38 #9212, p-P38 #9211, JNK #9258, p-JNK #4668 ; all from Cell Signaling Technologies, Beverly, MA; GAPDH #sc-47724 from Santa Cruz Biotechnologies, Santa Cruz, CA). The bands were visualized using horseradish peroxidase-conjugated secondary antibody (Amersham-Pharmacia Biotech, Piscataway, NJ), followed by enhanced chemiluminescence (Upstate, Waltham, MA) and documented by autoradiography (F-Bx810 Film, Phenix, Hayward, CA).

### Detection of intracellular ROS

Detection of intracellular ROS after treatment with non-complexed or Fe(III)- or Cu(II)- complexed salophene was measured by flow cytometry using carboxy-H2DCFDA dye (Invitrogen, Carlsbad, CA) as a probe. In the presence of a cellular oxidant, the compound produces green-fluorescence that is detected by flow cytometry. This dye detects the following ROS: hydrogen peroxide (H_2_O_2_), hydroxyl radical (HO^•^), and peroxyl radical (ROO^•^). SMS-KCNR (1.0×10^6^) cells were seeded into 100 mm^2^ cell and treated under the condition as indicated. Following treatment, cells were further incubated with 25 µM of carboxy-H2DCFDA for 30 min at 37°C with 5% CO_2_ in a humidified incubator. Cells were harvested by trypsinization, centrifuged, washed once with PBS and suspended in PBS. Data was acquired on a BD FACSort flow cytometer using CellQuest software (BD Immunocytometry Systems, San Jose, CA) and analyzed by using ModFit LT software (Verity Software House, Inc., Topsham, ME).
